# Diarrhœa—Podophyllum

**Published:** 1884-09

**Authors:** C. P. Jennings


### DIARRHŒA—PODOPHYLLUM.

C. P. Jennings, M. D.

April 23d (of this year), about 9 A. M., diarrhœa set in. It had been threatening for an hour or two, and at last hurried me to stool. Stomach had been disturbed through the night from the juice of canned plum. Stool dark-brown, mushy, copious, and attended with much wind. One or two stools in the after part of the day. Took no medicine. In the following night, about 3 A. M., driven out of bed suddenly. Stool watery, rather scanty, though gushing as if abundant. Stools became frequent. Occasionally a slight pain in abdomen. Much rumbling and gurgling. No thirst; no nausea. As the trouble began with indigestion, and this was brought on by fruit, I acted on general principles, and took Pulsatilla$^{200}$, one dose of a few pellets—half a dozen small, pin-head pellets. Waited four hours. Symptoms grew worse steadily. Acting still upon general principles, took Nux vomica$^{200}$, one dose. Repeated in three hours. Grew steadily worse. Three to six stools every hour; dark green, with yellow water; somewhat mealy; tenesmus; tendency to *prolapsus ani;* pains more frequent, and threatening to become intense and incisive and prolonged. Urine almost suppressed. Tongue moist, but beginning to put on a white coat and to be slimy. About noon of the 24th dissolved twelve small pellets of Podophyllum$^{200}$ in a glass of water. Of this solution took one teaspoonful. Within an hour symptoms became less urgent. By 6 P. M. they had nearly disappeared, and this without repeating the dose.

Podophyllum was indicated by the watery, mealy, dark-green stool, by the *prolapsus ani,* by the morning aggravation, and by the suppression of urine.

April 28th, a good stool. April 29th, 5 P. M., diarrhœa began as before; renewed at 8 P. M. Podophyllum$^{200}$, prepared as on the 24th. One teaspoonful sufficed. Up to this date, May 8th, no return of diarrhœa.

